# Wear Behaviors of the Surface of Duplex Cast Steel after the Burnishing Process

**DOI:** 10.3390/ma17081914

**Published:** 2024-04-21

**Authors:** Grzegorz Stradomski, Joanna Fik, Zbigniew Lis, Dariusz Rydz, Arkadiusz Szarek

**Affiliations:** 1 Faculty of Production Engineering and Materials Technology, Czestochowa University of Technology, 19 Armii Krajowej Av., 42-201 Czestochowa, Poland; dariusz.rydz@pcz.pl; 2 Faculty of Science and Technology, Jan Dlugosz University in Czestochowa, Armii Krajowej Street 13/15, 42-200 Czestochowa, Poland; j.fik@ujd.edu.pl; 3 Faculty of Mechanical Engineering, Bydgoszcz University of Science and Technology, 7 prof. S. Kaliskiego Av., 85-796 Bydgoszcz, Poland; zbigniew.lis@pbs.edu.pl; 4 Faculty of Mechanical Engineering and Computer Science, Department of Technology and Automation, Czestochowa University of Technology, 21 Armii Krajowej Av., 42-201 Czestochowa, Poland; arek@iop.pcz.pl

**Keywords:** wear, spalling, duplex cast steel, static pressure roller burnishing (SPRB)

## Abstract

Duplex steel and cast steels have a wide range of applications in many industrial sectors, for example, oil extraction, printing, petrochemical industry, energy—exhaust gases desulphurization systems, seawater desalination plants, and the shipbuilding industry. The machine elements can be produced with different techniques, which determine the operational properties. A material with the same chemical composition made as a casting will have worse mechanical properties than, for example, a forged element. This depends on the microstructure, its fragmentation and its morphology. However, the costs of casting are lower than, for example, forging, and, in addition, not all shapes obtainable in the casting process can be made using metal–plastic working methods. This article presents research results concerning the influence of the burnishing process on the properties of the duplex cast steel surface layer. The purpose of the research was to verify the impact of static pressure roller burnishing (SPRB) parameters on the wear of the surface layer of duplex cast steel. The subject of the research was cast steel in the GX2CrNiMoN22-5-3 grade—according to PN-EN 10283:2019—that was burnished using 15 variants of technological parameters. Then, the samples were subjected to surface wear tests using the INSTRON 8874 device. On the basis of the observed wear appearances, the acting wear mechanisms are defined and evaluated according their contribution to the wear behavior. Detailed information about the wear phenomena will help industries to minimize their maintenance losses related to surface wear. The possibility of shaping surface properties by mechanical burnishing is part of the current direction of surface engineering development. This technology, combined with a high-potential material such as duplex cast steel, makes it possible to increase wear resistance.

## 1. Introduction

The main area of application of ferritic–austenitic steels and cast steels, also known as duplex, constituting a group of corrosion-resistant steels, involves structures and elements exposed to high loads and environments conducive to stress, pitting or crevice corrosion. Using the well-known Schaeffler’s graph with the Ni and Cr equivalents helps to determine the share of individual components of a material’s microstructure. Very different properties, depending on the chemical composition and microstructure, constantly expand the areas of application of these materials. A special group of these materials is ferritic–austenitic steels, the development of which makes it possible to increase the durability of elements exposed to erosion and corrosion wear or the exploitation of deep-lying, highly sulfurized gas and oil deposits. Thanks to their high strength and plastic properties, they can be used on mining platforms and in deep wells, even at a depth of 8000 m, where the pressure reaches 1600 bar, and the temperature reaches 300 °C [1,2,3,4,5,6]. Some of the most important areas of application of ferritic–austenitic cast steel are pumps, valves, butterfly valves and other accessories used in many industries. These elements are most frequently produced in the form of castings and are often subjected to intense action of erosive and corrosive media, for example, in wet exhaust gas desulfurization and CO_2_ capture systems in the energy industry, oil and gas extraction or in hydrometallurgical processes. Due to different manufacturing methods, steel and cast steel products have significant differences in properties. Surface engineering is one of the areas of materials science that deals with the structure of materials in the surface layer of products, their modification and production processes, and the degradation of their microstructure components, leading to the wear of products during operation. Obtaining the desired operational effects of products is part of the current direction of scientific activities, which can be utilized in a wide range of applications [7,8,9,10]. Recently, there has been a noticeable increase in quality requirements in terms of dimensional accuracy and smoothness of products. Therefore, it is necessary to subject the manufactured components to additional surface finishing treatments. The use of finishing processing has a positive effect on the properties of products by improving their durability. There are many known methods of surface finishing, and among them is burnishing. The significance of the effect of the burnishing process depends on several factors, the main one of which is the method of preparing the surface before the burnishing process. The authors tested duplex cast steel, which after the casting process, was subjected to supersaturation in order to obtain a comparable volume share of the ferrite and austenite phases in the structure. In the next stage of material preparation, the external cylindrical surfaces were subjected to medium precision longitudinal turning. The turning process was carried out dry and without cooling, using a turning tool equipped with multi-edge inserts made of sintered carbides. The following cutting parameters were used during machining: feed f = 0.08 mm/rev; cutting depth an = 0.5 mm; rotation speed *n* = 1400 rpm; cutting speed vc = 190 m/min. The material prepared in this way was subsequently subjected to the static rolling pressure burnishing process. The possibility of shaping surface properties is part of the current direction of surface engineering development; it is also economically reasonable, but the resistance increase to fatigue and corrosion resistance have to be taken into consideration [7,8,9,10]. A potential area of application of machine and device elements subjected to the burnishing process is for massive castings of pump elements used in chemically aggressive environments. By increasing the smoothness, accuracy and hardness of the surface layer, it is possible to increase the service life of both the finished pump and its selected components.

This technology, combined with a high-potential material such as duplex cast steel, makes it possible to improve their functional properties [7]. At the outset, it should be emphasized that the mechanical treatment method presented in this study is well known in the industry, and research teams are currently intensively working on its development [11,12,13,14]. Burnishing technology is a method of finishing machining. The process of rolling static burnishing takes place in a machining system consisting of a machine tool, a holder, a tool and a workpiece. The process is carried out on cold (cold forming) and consists of achieving a local plastic deformation as a result of the applied burnishing tool and forces causing surface pressures exceeding the value of the plasticizing stress of the workpiece. In addition to removing unevenness, they also cause deformation of the surface layer of the workpiece. The effect of removing surface irregularities is a reduction in the roughness of the machined surface, while the effect of deformation is a fundamental change in the properties of the surface layer of the object [15]. As a result of the phenomena that occur during the burnishing process, the functional, physical, structural and mechanical properties and the stereometricity of the surface layer of the material are shaped. The surface condition obtained as a result of this process—a product’s functional properties—has a significant impact on the durability of manufactured metal products [16]. Obtaining high-quality products, which are characterized by resistance to wear, is one of the main goals of using friction nodes [17]. As indicated by B. Nowicki in [18], there is a strong correlation between roughness and fatigue strength.

It should be noted that not all materials can be burnished [12]. As indicated in the literature, duplex cast steel, despite its two-phase structure, can be burnished, and as a result, its surface properties can be shaped [19,20,21,22,23,24,25]. 

**Figure 1:**
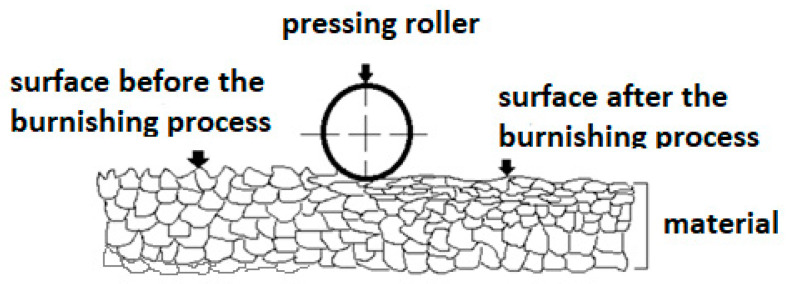
Process of structural changes in the material after the burnishing process.

The process of structural changes in the burnished zone is shown in Figure 1.

Wear is a real, practical problem in many industries. Abrasive wear alone has been estimated to cost 1–4% of the gross national product of industrialized nations [23]. For this reason, many studies aim to analyze this phenomenon in order to help industries to minimize their maintenance costs. The topicality of the issue is confirmed by numerous publications on contact fatigue [24,25,26,27,28]. The results presented in this article are a continuation and extension of this research.

## 2. Materials and Methods

The material used in this research was GX2CrNiMoN22-5-3 grade duplex cast steel—according to PN-EN 10283:2019 [29]. Samples were taken from cylindrical castings with a diameter of Ø45 mm. The castings were made using a remelting process, which was carried out in the metal melting laboratory of the Department of Metallurgy and Metal Technology of the Czestochowa University of Technology using the Leybold Heraeus IS1/III crucible induction furnace (Köln, Germany). Before the burnishing process, the material was heat-treated and machine-processed. The heat treatment involved a supersaturation process, and it was carried out at a temperature of approx. 1150 °C for 2 h to eliminate stresses, homogenize the microstructure and dissolve the intermetallic phases. After the supersaturation process, the machining process was carried out to obtain the final shape of the sample. With the sample shape used, it was possible that the burnishing process could be applied relatively easily. One of the very important advantages of this process (technique) is that there were no changes in the chemical composition of the product’s materials.

The chemical composition of the tested cast steel, summarized in Table 1, was established using the spectral method with the SPECTROLAB K2 spark spectrometer from Spectro (Kleve, Germany).

**Figure 2:**
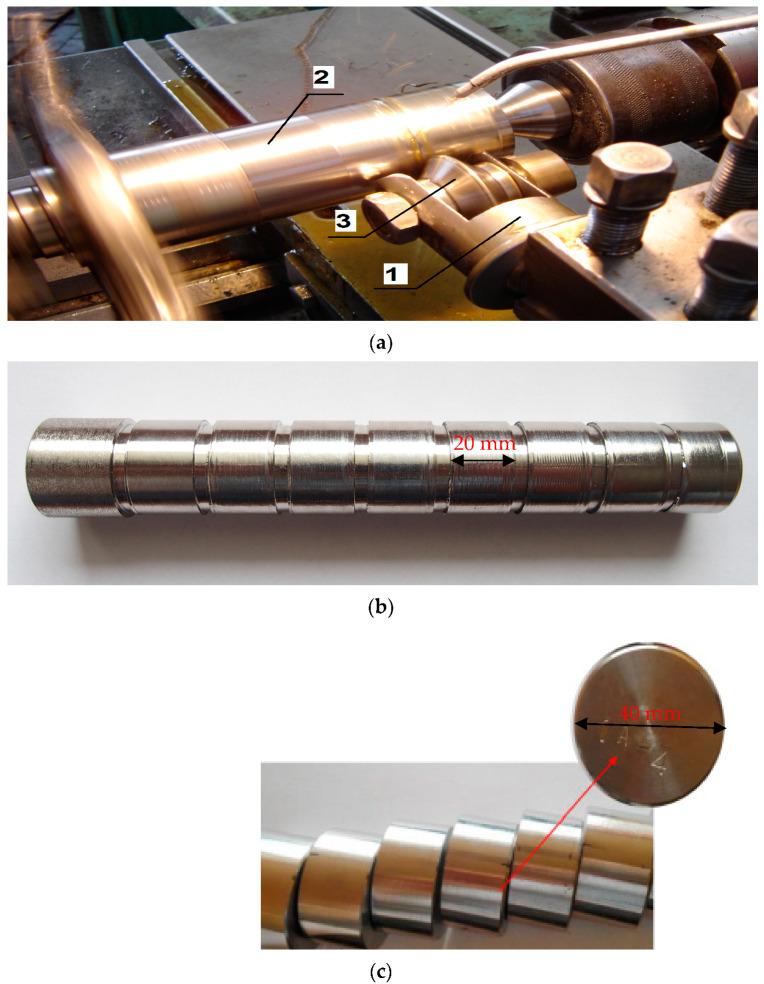
The schema of burnishing process (**a**): 1—burnisher roller (NK–01), 2—outer cylindrical surfaces, 3—disk burnishing tool; sample after the process (**b**); example of prepared marked samples (**c**).

Outer cylindrical surfaces were prepared for the burnishing process by machining on a universal lathe CDS 500 × 1000 with the following parameters: feed rate f = 0.08 mm/rev, depth of cut ap = 0.5 mm, cutting speed vc = 140 m/min. During the process, there was no cooling used, and the work carried out was dry. The technological parameters were selected on the basis of the authors’ own research and literature review [30,31,32,33,34]. The burnishing process was carried out on a laboratory stand, on a universal lathe, CDS 500 × 1000. The burner element was a disc-shaped roller burner (NK-01) with diameter of Ø50 mm and a rounding radius equal to 3 mm, made of 145CR6 tool steel with a hardness of 66 HRC (Figure 2).

The parameters of the technological process of burnishing was determined on the basis of preliminary experimental tests on materials with similar properties. The pressure force of the NK-01 burner was measured using a static method using the FT-5304M/A/16 strain gauge force sensor (INSTRON, Norwood USA). It was determined that a cut with a depth of 1 mm made by a burner is equivalent to the application of a force equal to 3 kN. In the process, machine oil was used for lubrication and cooling. The tests were carried out on three rollers. The process parameters for the variants are shown in Table 2. The main parameters were: feed rate—f_n_, burnishing depth—a_n_, total burnishing depth—a_nc_, number of passes—i, and burnishing speed v_n_, which was constant for all variants—100 mm/min.

**Figure 3:**
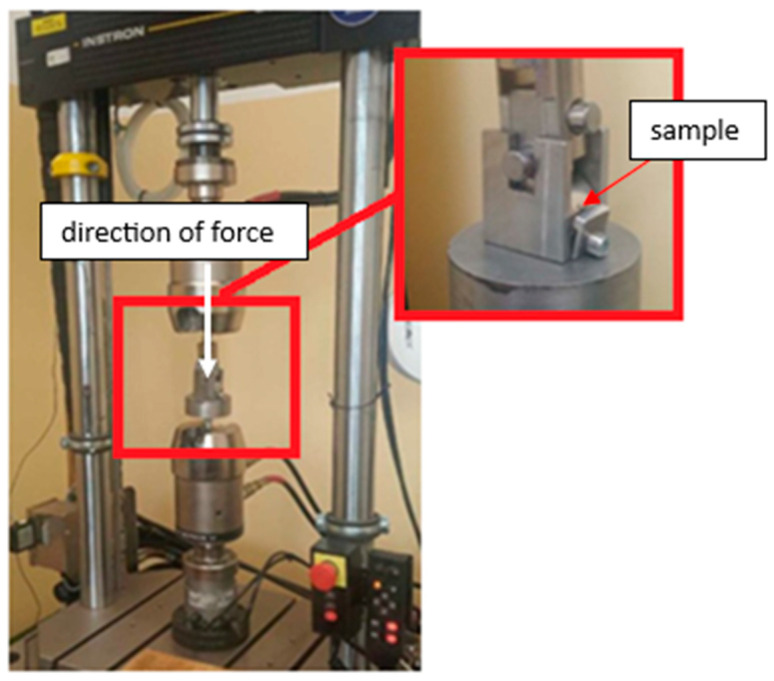
INSTRON 8874 device with the attached sample during the test.

The next stage of the conducted research, aimed at expanding the scope of knowledge on the operational properties of the tested materials, was testing the resistance to surface fatigue under cyclic impact load. The INSTRON 8874 testing machine (INSTRON, Norwood, MA, USA) was used for these tests (Figure 3). The analysis of the fatigue resistance of the material surface was carried out at a maximum clamping force of 2.2 kN. Other parameters that were used included: test frequency f = 20 Hz; sine cycle; a number of cycles equal to 3 million. The counter-sample was a cylinder with a diameter of 12 mm made of 100Cr6 bearing steel with a hardness of 58 HRC, which remained in constant contact and cooperated with the cylindrical surface of the tested materials. The value of the load was determined on the basis of preliminary tests carried out during the burnishing process.

**Figure 4:**
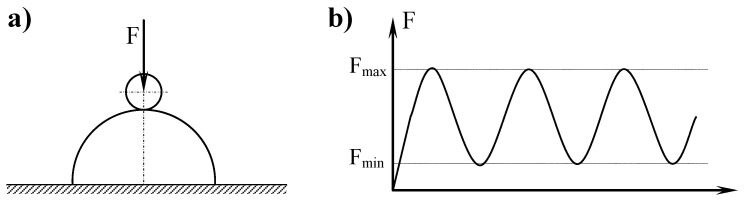
An illustrative image of the cooperating surfaces—(**a**), and a diagram of the action of the force over time—(**b**).

Figure 4 shows a scheme of the cooperating elements and a diagram of the force operation during the test.

**Figure 5:**
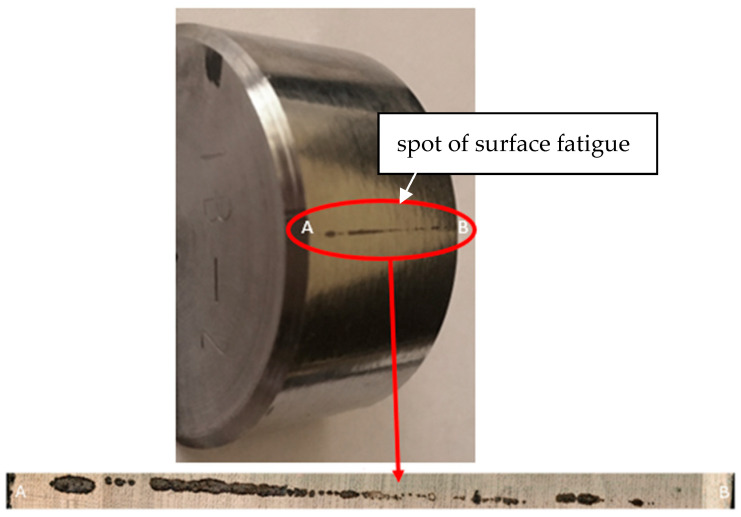
The sample used for the test with a visible spot of surface fatigue and macro image at 250× magnification.

The assessment of the fatigue life of the material was carried out under cyclic loads, with the appearance of cracks or chipping on the surface of the tested material as the criterion for failure. A quantitative assessment of structure damage, resulting from the development of processes causing material wear and the degradation of mechanical properties, was carried out by measuring the total area of the worn surface. In order to assess macro- and microstructure changes and to identify mechanisms that generate damage caused by cyclic sinusoidal loads, wear areas were observed using the KEYENCE VHX-7000 digital microscope (KEYENCE, Osaka, Japan) and the Phenom XL scanning microscope (Thermo Fisher Scientific Inc., Waltham, MA, USA). Figure 5 shows an example macroscopic image of the worn surface. To facilitate evaluation, the image below of the sample’s macro view shows a 250× magnification of the wear.

As can be seen, the geometry of the wear trace is not symmetrical. It can be assumed that this is the result of uneven microstructure precipitation. It should be remembered that the test material, i.e., duplex cast steel, contained a mixture of ferrite and austenite phases. The tested material had a share of both phases of 45/55% (ferrite/austenite). The share of phases was determined by quantitative metallography methods using Nis-Elements D software 3.20 (Nikon, Tokyo, Japan). The test itself was carried out in such a way as to eliminate potential random factors. Therefore, each counter sample used was single-use, and in order to eliminate the influence of differences resulting from the height, the fatigue test samples were ground with an accuracy of 0.01 mm.

## 3. Results

**Figure 6:**
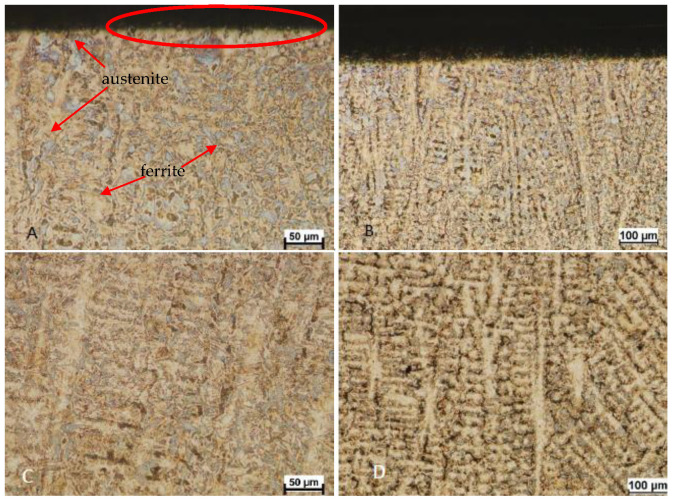
The microstructure of analyzed materials (**A**,**B**) surface layer (mag. 200× and 100×), (**C**,**D**) core of the tested castings (mag. 200× and 100×). Etched with the Mi21FE reagent.

The first stage of this research was carrying out microstructural studies, performed using a Nikon MA-200 optical microscope (Nikon, Tokyo, Japan). The material was etched using a Mi21Fe reagent (Figure 6). When analyzing the microstructure of materials such as ferrite–austenitic steels and cast steels, the choice of the etching reagent is of great importance. This is due to the fact that this material is characterized by a high tendency to produce intermetallic precipitates, often with destructive effects, such as the sigma phase. The proposed reagent allows for revealing the intermediate phases as well as the substituted phases. As can be observed in Figure 6, after the supersaturation process, there are no microstructure components other than ferrite and austenite.

The material is two-phase; it has a mixed microstructure of roughly equal proportions of austenite (white phase) and ferrite (Figure 6), but it is generally accepted that the expected properties can be achieved if ferrite and austenite are in a 30 to 70% ratio. As can be seen, the microstructure is typical for a cast with dendrites. Figure 6 shows the microstructure of the surface layer and the core. As can be seen, in both areas, the share of individual phases is similar and amounts to approximately 45/55% (ferrite/austenite). The microstructures visible in Figure 6 are shown at two magnifications: 100 and 200×. This method of presentation was intended to facilitate the observation of typical dendritic grains, clearly visible at 100× magnification. As can be observed, in the area of the surface layer, there is a surface deformation clearly visible at 200× magnification (area marked in red).

**Figure 7:**
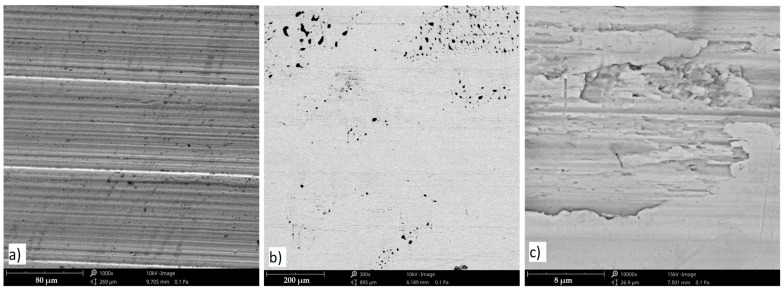
The surface of the tested material: (**a**) surface after machining process, (**b**) not loaded surface, (**c**) extrusions formed after long-term cyclic loads, scanning microscope—Phenom XL.

**Figure 8:**
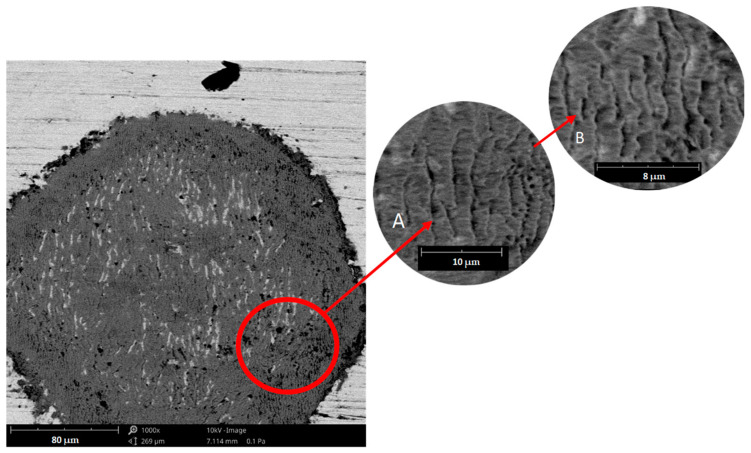
Periodic shifts on the surface of the tested material resulting from the action of a variable load—sample 4; (**A**) magnification 5000×; (**B**) area 10,000×, scanning microscope—Phenom XL.

Impact wear, the repetitive impact of two solid surfaces, causes material damage and removal. Under the influence of long-term cyclic loads, extrusions were observed on the surface of the tested material—Figure 7. Damages in the form of micro-cracks and chipping are shown in Figure 7 and Figure 8.

Based on the author’s own research and the literature data [35], it was found that the surface of the material shows signs of horizontal displacement of entire areas contained between slip bands, the development of which may be associated with the development of fatigue cracks—Figure 8.

**Figure 9:**
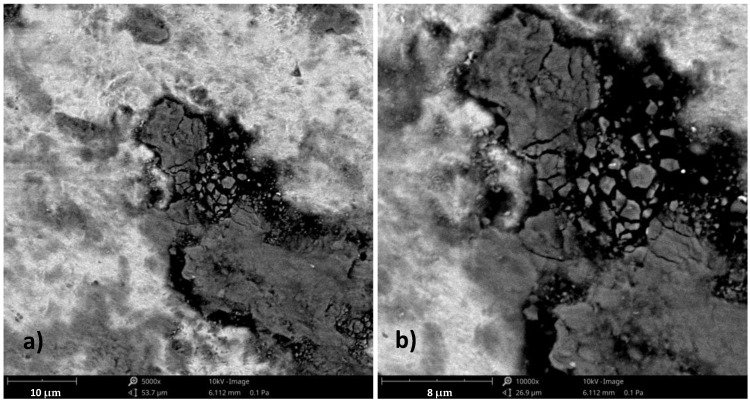
Microcracks and chipping on the surface of the tested material—sample 2; (**a**) magnification 5000×; (**b**) 10,000× magnification, scanning microscope—Phenom XL.

Damages in the form of micro-cracks and chipping are shown in Figure 9.

**Figure 10:**
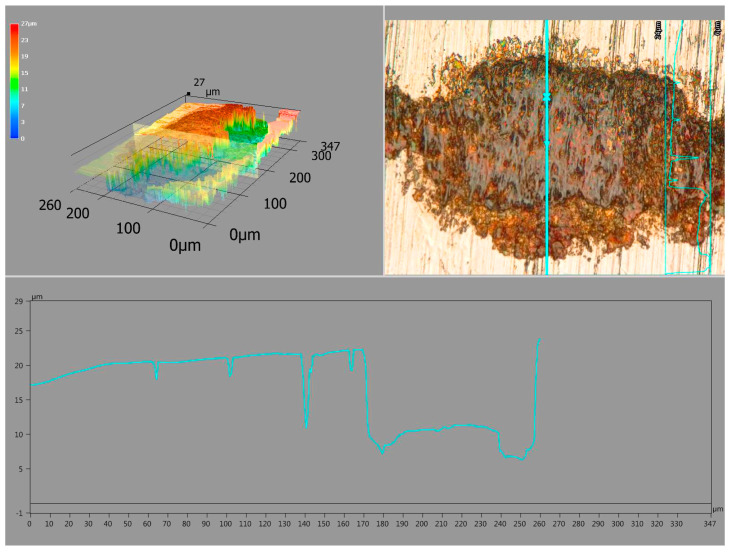
3D image of an example of chipping on the surface of the tested material—sample 2, KEYENCE VHX-7000 digital microscope.

After the analysis was carried out with use of SEM, 3D models were also made, along with the assessment of surface topography (Figure 10). Such tests allowed for a non-invasive assessment of the depth of the force impact during fatigue tests.

**Figure 11:**
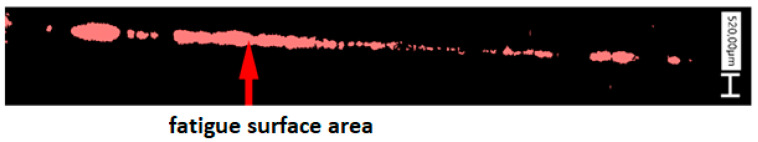
Sample fatigue surface area recorded using the Analyzer VHX 7000-970F software.

The surface area of material fatigue was used to assess the measure of damage. The measurement of the fatigue area was carried out using the KEYENCE VHX-7000 digital microscope (KEYENCE, Osaka, Japan) and the Analyzer VHX 7000-970F software (version 1.4.17.3) An example image of the measurements is shown in Figure 11.

**Figure 12:**
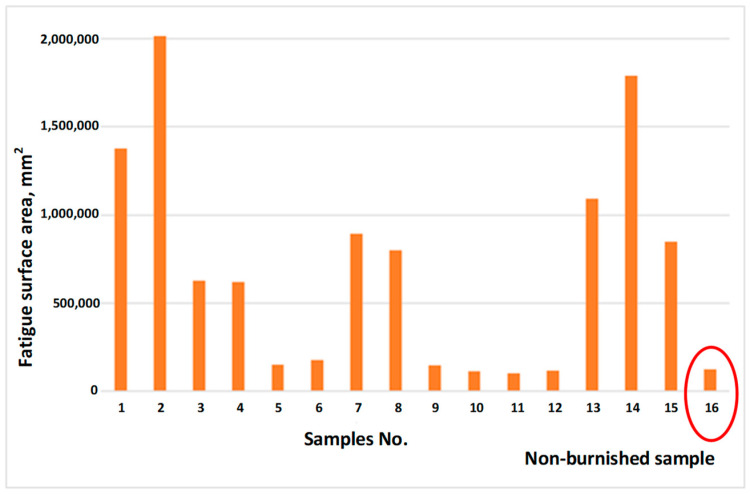
Graph comparing the worn surface area of individual samples.

The obtained results of fatigue surface area measurements, for all variants, are presented in Figure 12.

## 4. Discussion

The result of the burnishing process is the formation of the surface layer of the material with a zonal range. The range of changes and their degree depends on many factors, e.g., burnishing methods, process parameters, and the properties of the processed material. Many researchers, for example, [10,11,12,13,14,20,21,22,36,37], conduct research to determine the effect of this process on the properties of the surface of products whose surfaces cooperate with each other in cyclical contact; the resistance to contact fatigue is significant to the durability of a system. The surface damage created by cycle contact fatigue, as in the conducted research, has been given various names in the literature. As Tallinn points out [38], a one-millimeter crater, which is the final result of rolling contact fatigue, is named a spall and the damage process is called spalling. Other names used in the literature are pitting, flaking, micro-pitting and surface fatigue.

The analysis of the obtained results shows that the burnishing treatment of duplex cast steel GX2CrNiMoN22-5-3 makes it possible to shape the surface resistance to contact fatigue. The applied burnishing treatment parameters have a significant impact on the nature of the destruction of the surface layer. The material subjected to cyclic impact loading wears out due to a gradual increase in stresses around the defects in the structure of the surface layer of the associated elements. Then, microcracks are formed, which successively spread and cause spalling [38,39,40]. The observed geometry of the wear trace is not symmetrical. As was written earlier, the fatigue test samples were ground with an accuracy of 0.01 mm, and each counter sample was single-use. This was to prevent the problem of potential random factors. Since the sample preparation technique was very strict and the surface roughness after the burnishing process was very similar in all surface zones, it can be assumed that the wear trace geometry is connected with uneven microstructure precipitation. It should be remembered that the test material, i.e., duplex cast steel, contains a mixture of ferrite and austenite phases in its volume. Both phases have different plastic properties because of their crystallographic structures. The plastic and mechanical properties of duplex cast steel, which vary in volume, guarantee very good functional properties, and also determine changes in the surface layer. Since the burnishing process can be carried out using various techniques and in different ways, as presented in this work, it is important to examine their impact on parameters important from an operational point of view.

Depending on the parameters used for the burnishing process, it is possible to improve as well as deteriorate the fatigue life of a material. In connection with the above, it is important to properly select the parameters of the burnishing process. In connection to the experimental parameters used, the final effect of the burnishing treatment is significantly determined by the infeed depth of the burnishing tool a_n_. More favorable effects were obtained for samples that had a smaller depth of a single pass of the burnishing tool, amounting to 0.5 mm (total infeed 1 mm and two passes of the burnishing tool). In the case of using an infeed depth of the burnishing tool equal to 1 mm and one pass of the burnishing tool, the fatigue properties of both materials deteriorated compared to the reference sample, Figure 12 marked with red circle.

The main goal of this work, which was to indicate the possibility of using the burnishing process in cast products, was achieved. As research has shown, it is not only possible to shape the properties of the surface layer by deforming it, but it is also possible to obtain higher or at least the same resistance to surface fatigue. Determining the limit parameters is important with regard to operation and potential tribological wear, which during operation, is not only a derivative of the surface hardness but also its roughness and the resulting wear effects remaining in the friction node. The possibility of replacing higher-cost technologies with cheaper ones allows for optimization of the production process.

## 5. Conclusions

The research conducted here expands on the existing knowledge regarding the impact of burnishing treatment on the properties of castings made of duplex cast steel. The scientific studies available so far have focused mainly on materials produced in plastic-forming processes, and not, as in this case, on cast material. The conducted research confirmed the research hypothesis and achieved the assumed research goal. The key conclusion indicates that it is not only possible to burnish duplex cast steel castings, but also to perform the process in such a way that it is possible to obtain at least the same fatigue strength. Many authors, including [15,17,21], indicate in scientific studies that the technology in question may reduce fatigue resistance. Based on this research, the effect of improving the durability of components depends on the parameters of the burnishing process.

The conclusions resulting from the conducted research are as follows:

- The burnishing process can be used on duplex cast steel and deformation is observed on the surface layer;
- After the burnishing process, deformations are observed in both components of the microstructure—austenite and ferrite;
- The sample surfaces exhibited typical fatigue wear characteristic, and the wear mechanism of the surface depends on the parameters of the burnishing process;
- Burnishing of the tested duplex cast steel enables shaping of the contact fatigue resistance, so by burnishing, it is possible to increase the durability of a system consisting of elements whose surfaces cooperate with each other in cyclic contact;
- With regard to the applied parameters of the experiment, the final effect of the burnishing treatment is significantly determined by the infeed depth of the burnishing tool;
- Depending on the parameters used for the burnishing process, it is possible to improve (sample 10–12) or deteriorate the durability (sample 1, 2, 14) of the material, which is why the findings presented in this paper are crucial.

## Figures and Tables

**Table 1:** Chemical composition of the examined cast steel grade, weight %.

	C	Mn	Si	S	P	Cr	Ni	Mo	N	Fe
tested material	0.028	1.45	0.551	0.001 *	0.005 *	21.95	5.61	3.24	above 0.108	balance
according to the standard	0.03 *	2.0 *	1.0 *	0.025 *	0.035 *	21.0 to 23.0	4.5 to 6.5	2.5 to 3.5	0.12 to 0.2	balance

* maximum content.

**Table 2:** SPRB process parameters for tested variants.

Variants	1	2	3	4	5	6	7	8	9	10	11	12	13	14	15	16
f_n,_ mm/rev	0.1	0.2	0.3	0.4	0.5	0.6	0.1	0.2	0.1	0.2	0.3	0.4	0.5	0.1	0.2	non-burnished sample as a reference value
a_n_, mm	1	1	1	1	1	1	1	1	0.5	0.5	0.5	0.5	0.5	0.5	0.5	
a_nc,_ mm	1	1	1	1	1	1	2	2	1	1	1	1	1	1.5	1.5	
i [-]	1	1	1	1	1	1	2	2	2	2	2	2	2	3	3	

## Data Availability

Data are contained within the article.
